# Cost-effectiveness of Pharmaceutical-based Pandemic Influenza Mitigation Strategies^1^

**DOI:** 10.3201/eid1602.090571

**Published:** 2010-02

**Authors:** Anthony T. Newall, James G. Wood, Noemie Oudin, C. Raina MacIntyre

**Affiliations:** University of New South Wales, Sydney, New South Wales, Australia (A.T. Newall, J.G. Wood, C.R. MacIntyre); National Institute for Applied Sciences, Lyon, France (N. Oudin)

**Keywords:** Influenza, viruses, pandemic, mitigation, cost-effectiveness, vaccines, research

## Abstract

We used a hybrid transmission and economic model to evaluate the relative merits of stockpiling antiviral drugs and vaccine for pandemic influenza mitigation. In the absence of any intervention, our base-case assumptions generated a population clinical attack rate of 31.1%. For at least some parameter values, population prepandemic vaccination strategies were effective at containing an outbreak of pandemic influenza until the arrival of a matched vaccine. Because of the uncertain nature of many parameters, we used a probabilistic approach to determine the most cost-effective strategies. At a willingness to pay of >A$24,000 per life-year saved, more than half the simulations showed that a prepandemic vaccination program combined with antiviral treatment was cost-effective in Australia.

## Abstract 2

Population prepandemic vaccine and antiviral treatment strategies may be cost-effective.

Influenza pandemics of varying severity occurred 3 times in the last century (1918, 1957, and 1968); the first influenza pandemic of the 21st century occurred in 2009. Before this latest pandemic, awareness had been heightened by the emergence of the highly pathogenic (H5N1) strain (*1*). In response, many countries have developed detailed plans aimed at the mitigation of a future pandemic. A key aspect of many pandemic plans is the stockpiling of antiviral drugs (neuraminidase inhibitors) for treatment or prophylaxis (*2**,**3*).

The stockpiling of prepandemic vaccine is also an area of active consideration (*4*). Although a matched vaccine (developed specifically for the emergent strain) is likely to offer the best protection, the delay in producing such a vaccine is a major obstacle. The stockpiling of prepandemic vaccine based on currently available strains avoids this delay but such vaccine is likely to provide lower efficacy than a matched vaccine. There is also a substantial risk that the pandemic strain will be of a different subtype than that chosen for the stockpiled vaccine. The emergence of pandemic (H1N1) 2009 illustrates this point.

Mathematical models of disease transmission have been used to assess the feasibility of pandemic mitigation strategies (*5*–*10*). However, of the limited numbers of published economic evaluations on pandemic stockpiling (*11*–*14*), to our knowledge only 1 recent study has attempted to directly model herd protection (*14*). We explored the cost-effectiveness of stockpiling prepandemic vaccine and antiviral drugs for pandemic influenza mitigation.

## Methods

### Overview

**Figure 1:**
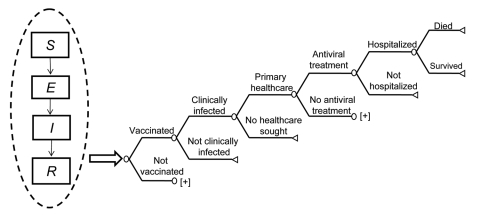
Schematic of hybrid transmission and decision analytic economic model. [+] indicates a cloned subtree with the same structure as the branch above. In sensitivity analysis, the probabilities of healthcare utilization and death were independent of each other but dependent on the probability of clinical infection. We assumed those with serious complications would seek primary healthcare. SEIR, susceptible, exposed, infected, removed.

An age-stratified transmission model (susceptible, exposed, infected, removed) was used to calculate clinical attack rates (CAR) and antiviral drug consumption, which became inputs in a decision analytic economic model as represented in Figure 1 (MATLAB version 2008a [www.mathworks.com]). The primary outcome from the economic model was the incremental cost per life-year saved (LYS). Economic results are reported per person in the population to facilitate understanding for an international audience. We addressed the uncertainty in many of the model parameters by performing extensive sensitivity analyses, including probabilistic sensitivity analysis using 5,000 Latin hypercube samples drawn from parameter distributions. A detailed description of the transmission model and a full list of model parameters and distributions can be found in the appendices (Technical Appendix 1, and Technical Appendix 2).

### Strategies

We considered prepandemic influenza vaccination in isolation and in combination with antiviral treatment. Four strategies for pandemic mitigation were examined (Table 1). In all strategies, a small stockpile of antiviral drugs was used for prophylaxis of case-patient contacts and treatment of clinical cases in an initial containment effort and, after a delay of 6 months, a matched vaccine was delivered. In isolation, this intervention was labeled strategy 1.

**Table 1:** Descriptions of 4 pharmaceutical-based pandemic influenza mitigation strategies*

Strategy no.	Description
1	Minimum pharmaceutical intervention
2	Antiviral treatment of those clinically infected
3	Population prepandemic vaccination
4	Strategies 2 + 3

*All strategies included an initial antiviral containment effort and distribution of a matched vaccine to the population 180 days after the first locally acquired case.

### Demographics

We divided the Australian population into 3 age groups: 0–19 years (26% [5,513,878], 20–64 years (61% [12,744,215], and >65 years (13% [2,759,129]) (*15*). Rates of mixing were age dependent and based on a recent large study of contact patterns in the European Union (*16*).

### Vaccine Parameters

Immunogenicity data provide evidence of prepandemic vaccine efficacy (VE) in humans (*17*). However, this efficacy will also depend on how closely the prepandemic vaccine strain matches the pandemic strain that emerges. We assumed that 2 doses of prepandemic vaccine would reduce susceptibility (relative hazard of infection) by 40% in persons <65 years of age. For the >65 years of age group, VE may be reduced (*18*). Thus, in our base-case model, we halved the VE for this age group. Modeled efficacy was also dependent on the number of doses, time since the last dose, and vaccine type (prepandemic or matched).

The first dose of prepandemic vaccine was assumed to be given coincident with the first local case-patient, followed 21 days later by the second dose. In all strategies, the first dose of matched vaccine was provided 180 days after the first local case-patient was identified in Australia; the second dose was administered 21 days later in strategies 1 and 2 only. Although 2 doses of a matched vaccine would be ordered under all strategies, in base-case only 1 dose of matched vaccine was administered in strategies involving 2 doses of a prepandemic vaccine (strategies 3 and 4). Population vaccine coverage (both prepandemic and matched) was assumed to be 80% (*19*).

### Antiviral Drug Parameters

In the base-case model, we estimated the efficacy of antiviral treatment for preventing hospitalization as 59% (*20*). We assumed the same antiviral drug efficacy for preventing death as for hospitalization. The effect of antiviral treatment on influenza transmission in the community is unclear (*21*), and we assumed no reduction in infectivity of treated case-patients. When antiviral treatment was given, it was provided to 80% of persons with clinical disease (those who sought primary healthcare). Antiviral drug strategies contained stockpiles to cover 40% of the population (≈8 million courses). However, all strategies assumed a limited antiviral drug stockpile (≈0.2 million courses) to be used in initial containment efforts for treatment of clinical case-patients and prophylaxis of case-patient contacts. We assumed that the antiviral prophylaxis treatment of contacts, during the initial containment effort, reduced susceptibility and infectiousness by 70% and 60%, respectively (*22*). A static percentage (10%) of viruses were assumed resistant to antiviral drugs.

### Disease Estimates

The CAR during a pandemic was determined by using the transmission model and was primarily a function of *R*_0_ (1.7) (*23*–*25*) and the percentage of asymptomatic infections (50%) (*26*). The basic reproductive number (*R*_0_) represents the number of secondary case-patients that a representative person with influenza would infect in a fully susceptible population. Asymptomatic persons were assumed to be two thirds as infectious as symptomatic persons (*9**,**10*). We assumed that 50% of those with a clinical influenza infection would seek medical care (*27*); most primary care would occur in general practice (80%) and the remainder in hospital emergency departments.

The rates of hospitalization and death were defined relative to the CAR by using a patient-hospitalization rate and patient-fatality rate. We used age-specific case-hospitalization rates (0–19 years = 1.875%, 20–64 years = 2.5%, >65 years = 5%) and patient-fatality rates (0–19 years = 0.75%, 20–64 years = 1%, >65 years = 2%). There are no reliable estimates of when a future pandemic might occur. We used a base-case delay to a pandemic of 5 years.

### Cost Estimates

As recommended by Australian pharmaceutical funding guidelines, we focused on direct healthcare costs (*28*) and performed our base-case analysis from a healthcare system perspective. In scenario analysis we considered a broader societal perspective, which included lost production costs. Costs and effects were discounted at 5% annually (*28*). All costs are reported in 2005 Australian dollars.

#### Intervention Costs

In the base-case model, we assumed a stockpile purchase price for pharmaceuticals of $12 per vaccine dose and $32 per antiviral course; a range of values was considered in sensitivity analysis. The limited shelf-life of the pharmaceuticals requires the renewal of stockpiles for prepandemic vaccine every 3 years and antiviral drugs every 5 years. The number of times stockpiles were replaced was based on the expected time to a pandemic. We assumed partial replacement of the stockpile annually on a continuous basis. An annual storage cost for vaccines ($1, refrigeration) and antiviral drugs ($0.5, no refrigeration) was included.

We assumed that vaccination (and initial antiviral drug distribution) would be administered in mass clinics at a cost of $11.60 per course/dose (*29*). An administration cost for antiviral treatment was not included because this treatment would be given as part of a primary care visit for influenza illness. However, for strategies that included antiviral treatment, the percentage of clinical case-patients seeking medical care was increased to 80%.

#### Healthcare Unit Costs

Hospitalization costs were based on analysis previously conducted by our group, which reviewed records of patients hospitalized for influenza and pneumonia in Australia (*30*). We estimated age-specific hospitalization costs by multiplying the average cost per day by the average length of stay for that age group (*31*). Expenses for emergency department visits for influenza not requiring hospitalization were estimated by the Australian Ambulatory Classes emergency department presentation cost for “Other respiratory diseases with procedure” (*32*). The cost of a general practitioner visit for influenza ($33.32) was based on a general practitioner survey of consultation for influenza-like illness (*30*).

#### Production Costs (Societal Perspective Only)

The costs of lost production were valued by using the human capital approach. Lost production was only valued for those employed in paid work (*33*). The cost attached to lost work days was based on average weekly earnings (*34*). Clinical influenza patients were assumed to have 2.6 days absent from work (*35*). We assumed that those <15 years of age would require 1 adult caregiver when sick. We used length of stay to estimate lost production for hospitalized patients.

## Results

### Clinical Outcomes

The base-case analysis used an *R*_0_ value of 1.7, which led to a CAR of 31.1% in the overall population in the absence of any intervention. The assumption of greater mixing in children meant that this group experienced the highest CARs, with 38.1% in persons 0–19 years of age, 30.4% in persons 20–64 years of age, and 20.4% in persons >65 years of age. In the absence of any intervention, the base-case model produced an overall population hospitalization rate of 782.3/100,000 persons and a mortality rate of 312.9/100,000. Strategies incorporating a population prepandemic vaccination program resulted in a low CAR and, consequently, a low number of hospitalizations (strategy 3 = 136.8/100,000; strategy 4 = 79.4/100,000) and deaths (strategy 3 = 54.7/100,000; strategy 4 = 31.8/100,000). The antiviral drug treatment strategy did not affect the CAR but significantly reduced the number of hospitalizations (strategy 2 = 450.0/100,000) and deaths (strategy 2 = 180.0/100,000).

**Figure 2:**
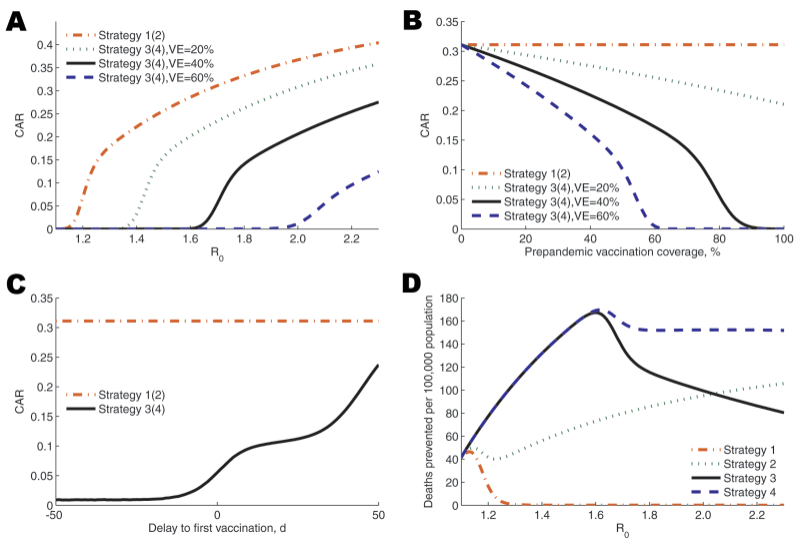
Sensitivity analyses of clinical outcomes as key parameters are varied. In A–C, the clinical attack rate (CAR) is displayed as a function of *R*_0_ and vaccine efficacy (VE) (A), vaccine coverage and VE (B), and the delay to vaccination (C). In D, deaths prevented per 100,000 population compared with no intervention is displayed as a function of *R*_0_.

Several parameters were influential in determining the CARs (Figure 2). In prepandemic vaccination strategies, *R*_0_ (Figure 2, panel A), VE (Figure 2, panel A), and vaccine coverage (Figure 2, panel B) played major roles in determining whether a large outbreak was prevented or simply mitigated. The CAR rose as *R*_0_ increased and declined as VE improved and coverage increased, with sharper transitions occurring as the number of secondary case-patients that a single case-patient infects approached 1. The CAR for prepandemic vaccination strategies also increased markedly when vaccination was delayed until after a local outbreak had commenced (Figure 2, panel C). The number of deaths prevented by antiviral treatment rises as *R*_0_ increases (Figure 2, panel D). This increase occurs because the incidence of preventable disease is larger for higher values of *R*_0_. The effect on prepandemic vaccination strategies is similar, provided the strategy is largely successful in containing the pandemic. However, for *R*_0_ values >1.7, when this is no longer the case, prepandemic vaccinations strategies prevented fewer deaths (Figure 2, panel D).

### Economic Outcomes

The total discounted healthcare costs for a pandemic in the absence of any intervention was $31.1/person in the population. The gross discounted cost over 5 years (including purchase, replacement, storage, and administration) of a prepandemic vaccination program was $68.4/person in the population and the cost of an antiviral treatment program (purchase, replacement, and storage only) over the same period was $24.8/person.

The base-case results for the healthcare system perspective are shown in Table 2. In the base-case model (*R*_0_ = 1.7, VE = 40%), strategies 2–4 each offered increased effectiveness at an increased cost when compared with the next best strategy. Under these conditions (theoretically), decision makers should first decide if strategy 2 offers value for money (incremental cost-effectiveness ratio [ICER] = $909/LYS) and then consider the value offered by each additional increase in spending, moving from strategy 2 to 3 (ICER = $1,084/LYS) and then from strategy 3 to 4 (ICER = $7,458/LYS).

**Table 2:** Base-case economic results per person in the population (healthcare system perspective) of 4 pharmaceutical-based pandemic influenza mitigation strategies*

Strategy no.	Net cost	Incremental cost	LYS	Incremental LYS	Incremental cost per LYS†
1	65.88	–	–	–	–
2	82.24	16.36	0.01803	0.01803	908
3	100.65	18.40	0.03501	0.01698	1,084
4	124.00	23.36	0.03814	0.00313	7,458

*LYS, life-year saved. Costs and life-years discounted at 5% annually; all costs calculated in 2005 Australian dollars. †Rounded to the nearest whole dollar.

From a societal perspective the least costly strategy was prepandemic vaccination (strategy 3), which was cost saving when compared with the minimum pharmaceutical intervention. Strategy 3 also dominated antiviral drug treatment alone (strategy 2), being more effective and less costly. The addition of antiviral drug treatment to prepandemic vaccination cost $7,404/LYS.

#### Sensitivity Analyses (Healthcare System Perspective)

Key parameters affecting the cost-effectiveness of strategies included the *R*_0_ value and factors impacting vaccine or antiviral effectiveness. Because strategies differed in their sensitivity to these parameters, the cost-effectiveness of strategies relative to each other varied. Dominance occurs when a strategy is considered superior to the alternative by being either more effective and less costly (simple dominance) or more effective and more costly but with a lower ICER (extended dominance) (*36*). At higher values of VE (>41%) or when the percentage of antiviral given within 48 h was <75% (base-case = 80%), prepandemic vaccination dominated antiviral drug treatment alone. When the VE was >50% or the *R*_0_ <1.6, prepandemic vaccination alone was largely sufficient to contain the pandemic, and the addition of antiviral treatment offered only a minimal incremental effect at a high incremental cost (ICER >$1million per LYS). At lower values of VE (37%) or at higher values of *R*_0_ (1.8), prepandemic vaccination alone was dominated by prepandemic vaccination combined with antiviral drug treatment, which offered reasonable value for money (ICER <$3,500/LYS) when compared with antiviral drug treatment alone. When we considered a VE of 20%, the addition of prepandemic vaccination to antiviral treatment alone cost ≈$9,000/LYS.

To be cost saving, prepandemic vaccine (strategy 3) and antiviral drug treatment (strategy 2) would have to be priced at <$3.1/dose and <$10.0/course when compared with the minimum pharmaceutical intervention. Variation in most other parameters did not affect the cost-effectiveness of strategies relative to each other. When the CAR was reduced (20% in the absence of any intervention) as a result of the percentage of asymptomatic infections, the ICER of all strategies increased. However, all strategies still had an ICER <$10,000/LYS. When we assumed the pandemic was relatively mild (patient-fatality and patient-hospitalization rates 5× less than base-case) and occurred 30 years later, all strategies had ICERs >$50,000/LYS. Varying the age distribution of severe clinical case-patients (patient-fatality and patient-hospitalization cases) had only a minor impact on the cost-effectiveness. When we varied the discounting rate (to be either 0% or 3% for costs and effects), ICER for all strategies decreased with no change to strategy order. Variation in other parameters was explored in probabilistic sensitivity analysis.

#### Probabilistic Sensitivity Analyses

**Figure 3:**
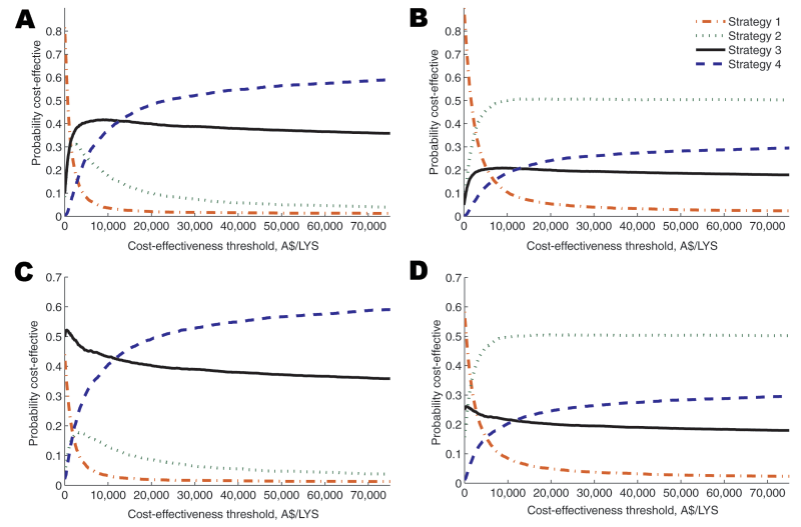
Cost-effectiveness acceptability curves. Panels A and B show the healthcare system perspective; C and D show the societal perspective. In B and D, we assumed that half of the time (Q = 50%) the emergent pandemic strain would be would be of a subtype to which the stockpiled vaccine offered no protection. We did not explore the use of such a vaccine in subsequent pandemics. Costs and life-years discounted at 5% annually. A$, Australian dollars; LYS, life-year saved.

Cost-effectiveness acceptability curves (Figure 3) enable decision makers to estimate the probability that a strategy is optimal as a function of their willingness to pay for additional units of effect. At decision makers’ willingness to pay >$24,000/LYS, more than half of simulations found that a prepandemic vaccination program combined with antiviral treatment was cost-effective in Australia (Figure 3, panels A and C). However, when we assumed that half of the time the emergent pandemic strain would have a different subtype than that chosen for the stockpiled vaccine (Figure 3, panels B and D), most simulations (willingness to pay >$12,000/LYS) found that antiviral drug treatment alone was the optimal strategy.

## Discussion

Under the assumption of a severe pandemic occurring in the near future, the pharmaceutical-based mitigation strategies examined were generally estimated to be cost-effective. For at least some of the plausible range of transmission parameters, strategies involving population prepandemic vaccination were effective in containing an outbreak until the arrival of a matched vaccine. A combination of antiviral drug treatment and prepandemic vaccination offered the best protection for the population. From a societal perspective, prepandemic vaccination was estimated to be cost saving when compared with the minimum pharmaceutical intervention.

The cost-effectiveness of pandemic influenza mitigation strategies was quite resilient to major changes in influential parameters such as the value of *R*_0_ and the effectiveness of vaccination and antiviral drugs. This resilience stems from 2 important assumptions: 1) we assumed that the pandemic would be severe (our base-case has similar characteristics to the 1918 pandemic); and 2) we assumed a pandemic would occur soon (5-year delay in base-case). The first assumption implies that the consequences of a pandemic would be large in terms of the number of deaths and the healthcare resources required, whereas the second assumption implies that the costs associated with maintaining a stockpile were limited and that the future benefits would not be dramatically reduced by discounting. Under these assumptions, even moderately effective interventions from a clinical perspective (e.g., a vaccine with 20% efficacy) may be cost-effective. When we assumed instead that the pandemic was relatively mild (patient-fatality and patient-hospitalization rates 5× less than base-case) and occurred 30 years later, pandemic mitigation strategies were borderline cost-effective at best. This mild scenario still assumes a disease incidence several times that of seasonal influenza.

We found that vaccination and antiviral strategies differed in their sensitivity to certain key parameters (Figure 2). Because the value for value for money offered by the intervention strategies was relatively similar, even minor changes in some parameters led strategies to become dominated by a more effective alternative. For example, when VE increased above 41% (base-case 40%), antiviral drug treatment alone was dominated (extended) by prepandemic vaccination (strategy 3). This sensitivity highlights the inadequacy of a base-case analysis and the need for probabilistic sensitivity analysis (Figure 3).

This analysis was restricted by a lack of accurate information on prepandemic VE. However, because any emergent pandemic strain is unknown, some level of uncertainty around VE is unavoidable. We assumed that a prepandemic vaccine would offer moderate protection (below that of a matched seasonal vaccine), and using probabilistic sensitivity analysis (Figure 3, panels B and D), we explored the risk that the emergent pandemic strain would be of a different subtype than that chosen for the stockpiled vaccine. We did not specifically explore the use of such a vaccine in subsequent future pandemics or the separate stockpiling of adjuvant and antigen. The effectiveness of antiviral drugs can also not be known with certainty in advance. We assumed a static 10% resistance to antiviral drugs and varied this widely in sensitivity analysis. A more realistic model would take into consideration possible development of resistance over time (*37*), but a detailed analysis of antiviral resistance was beyond the scope of our analysis.

Our model approach was deterministic so that although stochastic variation in parameters was considered, identical parameter choices led to identical model outputs. Because our analysis was limited to assessing the effect on overall attack rates and the costs and benefits associated with this, rather than outputs such as daily case counts, the influence of stochasticity at the simulation level should be relatively minor. Furthermore, the importation of cases from outside the country is likely to rapidly increase counts to a level at which deterministic behavior dominates. A major advantage of a simple deterministic approach is that sensitivity analyses are not constrained by computational resources, enabling detailed uncertainty analysis.

We have largely ignored issues of capacity constraint. For instance, hospital bed day capacity is likely to be severely strained during the peak of an influenza pandemic (*38*). A severe influenza pandemic is also likely to have a dramatic effect on the broader economy (*39*), which may not be captured well even under our societal perspective. Studies estimating the macroeconomic impact of a pandemic are beginning to emerge (*40*). The failure to capture the broader macroeconomic impact makes our healthcare system perspective conservative. However, the extent to which the benefits are captured (or not captured) is likely to be different for each strategy.

Population prepandemic vaccine and antiviral drug treatment strategies offer substantial scope to be cost-effective strategies for pandemic influenza mitigation. Unlike antiviral treatment strategies, population prepandemic vaccination offers the possibility of containment until the arrival of a matched vaccine. The stockpiling of prepandemic vaccines should be carefully considered and take into account the current level of uncertainty and budgetary limitations.

## Supplementary Material

Technical Appendix 1 Description of the transmission model

Technical Appendix 2 Parameters: base-case and sensitivity range
