# Supplementary material for: Cost-effectiveness of Pharmaceutical-based Pandemic Influenza Mitigation Strategies
Source: Emerg Infect Dis. 2010 Feb;16(2):224–30. doi: 10.3201/eid1602.090571 (PMC2957998; doi:10.3201/eid1602.090571)
Supplement: Technical Appendix 1 — Description of the transmission model [file 09-0571_Techapp1-s1.pdf]

Cost-effectiveness of Pharmaceutical-based Pandemic Influenza Mitigation Strategies

Technical Appendix 1. Description of the transmission model

Model structure

The model was structured as a set of "Susceptible, Exposed, Infected, Removed" (SEIR)-type deterministic differential equations:

$$\frac{d\vec{S}}{dt} = -\vec{\lambda}.*\vec{S}$$

$$\frac{d\vec{E}}{dt} = \vec{\lambda}.*\vec{S} - \omega\vec{E}$$

$$\frac{d\vec{I}}{dt} = \omega\vec{E} - \gamma\vec{I}$$

$$\frac{d\vec{R}}{dt} = \gamma\vec{I}$$

The states are vector structures containing 12 elements representing 3 possible vaccination groups (no-vaccine (i=1), pre-vaccine and matched vaccine (i=2) or matched vaccine only (i=3)) and 3 population groups (0-19 years (j=1), 20-64 years (j=2), and 65+ (j=3)). Susceptible states, for example, can be written as S_i^j, i,j=1..3. The parameters ω and γ were fixed constants representing the rate of progression from exposure to becoming infectious and the rate of recovery from infection respectively. Births and deaths were assumed to make a negligible contribution over the time-scale of the epidemic. We also assumed that there are a growing number of imported cases during the early stages of the pandemic (discussed below). See Appendix 2 for parameter values and ranges.

The force of infection vector $\vec{\lambda}$ was dependent on several time-dependent factors including the prevalence of infection and the timing of vaccination programs. We defined $\vec{\lambda} = \hat{\beta}\vec{I} / N$, where $\hat{\beta}$ is the who-acquires-infection-from-whom (WAIFW) matrix and N was the

population size. The mixing between population groups without considering interventions was defined as:

$$\hat{\beta}_0 = \begin{pmatrix} \beta_{11} & \beta_{12} & \beta_{13} \\ \beta_{21} & \beta_{22} & \beta_{23} \\ \beta_{31} & \beta_{32} & \beta_{33} \end{pmatrix}$$

Mixing Matrix

The matrix $\hat{\beta}_0$ was calculated using data from a recent POLYMOD survey of contacts conducted in the European Union (*1*). In order to construct our matrix, we first calculated the (unweighted) average of the matrices for close contacts over all countries. We then reduced this to a 3x3 matrix describing contacts between 0-19, 20-64 and 65+ age groups by taking the average over the relevant sub-matrices. This involves some loss of fidelity, as the POLYMOD data is stratified into 5 year age bands for individuals under 70 years of age. The base-case contact matrix is then given by

$$\begin{pmatrix} \beta_{11} & \beta_{12} & \beta_{13} \\ \beta_{21} & \beta_{22} & \beta_{23} \\ \beta_{31} & \beta_{32} & \beta_{33} \end{pmatrix} = \frac{R_0}{M} \begin{pmatrix} 1.4 & 0.35 & 0.18 \\ 0.39 & 0.49 & 0.26 \\ 0.18 & 0.23 & 0.6 \end{pmatrix}$$

which involves a scaling factor R_0/M , so that the the next generation matrix **R** has maximum eigenvalue of R_0:

$$\mathbf{R}_{ij} = \beta_{ij}\pi_i, \quad M = \max eig \begin{pmatrix} \beta_{11}\pi_1 & \beta_{12}\pi_1 & \beta_{13}\pi_1 \\ \beta_{21}\pi_2 & \beta_{22}\pi_2 & \beta_{23}\pi_2 \\ \beta_{31}\pi_3 & \beta_{32}\pi_3 & \beta_{33}\pi_3 \end{pmatrix}, \quad i, j = 1..3$$

Here π_i is the proportion of the population in the ith age class. The final mixing matrix involving pharmaceutical effects is expressed as the element by element matrix product $\hat{\beta} = \hat{\beta}_v .* \hat{\beta}_a$, where $\hat{\beta}_v$ incorporates vaccine effects and $\hat{\beta}_a$ incorporates antiviral effects.

Clinical attack rates

Clinical attack rates (CARs) are calculated in the model as the percentage of the population who became infected during the course of the epidemic multiplied by the proportion of infections that are clinical (50% in base-case). CARs are sensitive to the structure of the mixing matrix – the assortative nature of mixing predicted by the POLYMOD data (*1*) means

that attack rates for a given R_0 are lower than if mixing is uniform. In Table S1 below, we compare base-case population CARs from the above mixing matrix and a uniform mixing matrix by strategy. In all cases the attack rate is higher under the assumption of uniform mixing with the largest difference in both absolute and relative terms occurring for whole of population pre-pandemic vaccination strategies. However, by using broad age classes we have underestimated the level of assortativity in mixing, so it is likely that our base-case matrix underestimates the protective effect of the intervention.

Table: Comparison of Clinical attack rates (CARs) derived from the base-case mixing matrix with those from a uniform mixing matrix

**Strategy**	**CAR (base-case matrix)**	**CAR (uniform matrix)**
Strategy 1(2)	31.1%	34.56%
Strategy 3(4)	5.53%	12.14%

Effect of the vaccine

The vaccine was assumed to reduce susceptibility only (with no further impact on disease or infectiousness of breakthrough cases), while antiviral prophylaxis was assumed to be both protective against infection and to reduce infectiousness of individuals with breakthrough infection. Full efficacy is achieved 21 days after vaccination with protection rising along a logistic curve in between vaccination and this time point. This process is repeated for each dose and protection is assumed not to wane. The efficacy of the vaccine in those over 65 years of age was assumed to halve the efficacy in the younger age-groups. The base-case evolution of vaccine efficacy is shown in the Figure.

Figure. Schematic of vaccine efficacy over time in base-case analysis

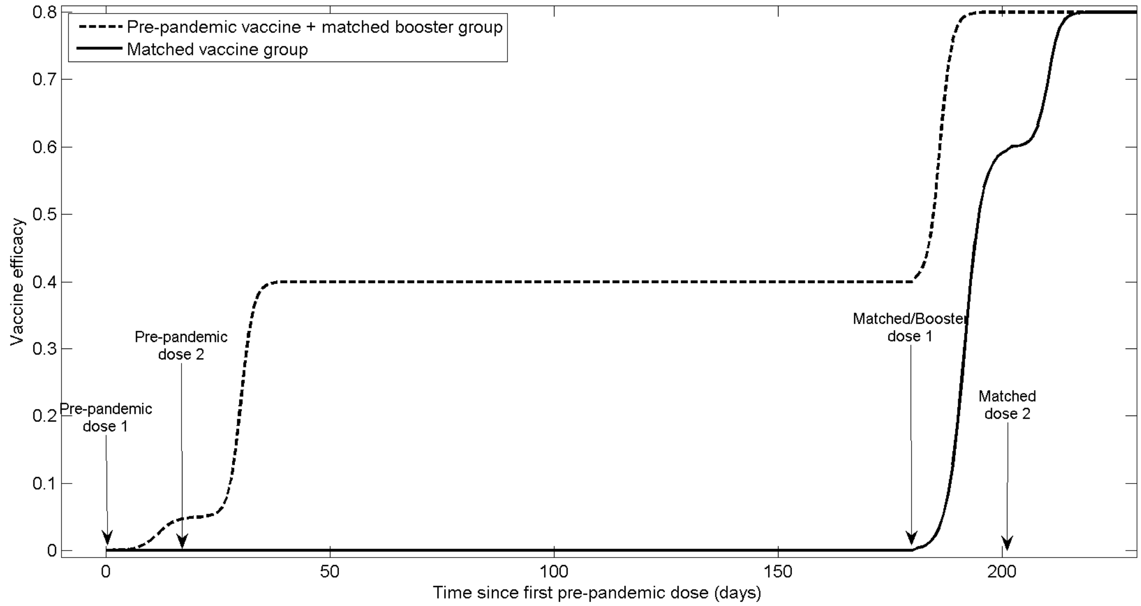

Delivery of the vaccine to the target population is assumed to occur instantaneously. In practice, this means the interpretation of vaccine timing should be as the midpoint of the vaccination campaign (i.e. when vaccine has reached 50% of the target group). Doses of the pre-pandemic or matched vaccines are given 21 days apart.

Effect of antiviral drugs

Antiviral drugs were used for both treatment and prophylaxis, with treatment assumed to affect only the risk of hospitalisation and death following infection (but not the infectiousness of a treated case), whereas prophylaxis was assumed to reduce the risk of infection and the infectiousness of breakthrough cases. We made no distinction between pre and post exposure prophylaxis in terms of efficacy. However, it was assumed that only a limited proportion of case contacts (30% in base-case) would receive prophylaxis in time to achieve an effect. We assumed there was no difference in efficacy by age or vaccination status.

The effectiveness of antiviral drugs was reduced by the presence of resistant virus. A constant level of resistance was applied (10% in base-case) and antiviral drugs were assumed to have no efficacy against resistant virus. This approach ignores the likely dynamic competition between resistant and sensitive strains explored elsewhere (*2*,*3*).

Depletion of the antiviral drug stockpiles depended on what intervention the stockpile was used for. When used for post-exposure prophylaxis of case contacts, 100 antiviral drug courses were assumed to be dispensed for each symptomatic case; for treatment, one antiviral course was used for each treated case. The use of antiviral drugs for treatment and prophylaxis was in accordance with licensing guidelines. The distribution of antiviral drugs for prophylaxis is likely to over-estimate use but is based on the assumption that prophylaxis would be offered to a large number of people for each case identified at the start of an epidemic. This parameter has very little influence on model outcomes.

Sub-clinical Infection

In the base-case analysis we assumed that half of all infections were asymptomatic to mildly symptomatic (sub-clinical) and would not present to health care providers. This was varied in sensitivity analysis and was an influential variable since it directly affects the clinical attack rate. Infectiousness of sub-clinical cases was set to two-thirds of that of clinical cases (range 33%-100%) but was not an influential variable.

Imported Cases

We assumed that imported cases would make an important contribution to the epidemic in the early stages. In the first week, cases were assumed to arrive at a rate of 1 per day, with this rate of importation doubling each week for 4 further weeks and then being sustained for a further 5 weeks.

References

1. Mossong J, Hens N, Jit M, Beutels P, Auranen K, Mikolajczyk R, et al. Social contacts and mixing patterns relevant to the spread of infectious diseases. PLoS Med. 2008;5:e74. PubMed DOI: 10.1371/journal.pmed.0050074

2. Lipsitch M, Cohen T, Murray M, Levin BR. Antiviral resistance and the control of pandemic influenza. PLoS Med. 2007;4:e15. PubMed DOI: 10.1371/journal.pmed.0040015

3. McCaw JM, Wood JG, McCaw CT, McVernon J. Impact of emerging antiviral drug resistance on influenza containment and spread: influence of subclinical infection and strategic use of a stockpile containing one or two drugs. PLoS One. 2008;3:e2362. PubMed DOI: 10.1371/journal.pone.0002362
