# Supplementary material for: Cost-effectiveness of Pharmaceutical-based Pandemic Influenza Mitigation Strategies
Source: Emerg Infect Dis. 2010 Feb;16(2):224–30. doi: 10.3201/eid1602.090571 (PMC2957998; doi:10.3201/eid1602.090571)
Supplement: Technical Appendix 2 — Parameters: base-case and sensitivity range [file 09-0571_Techapp2-s2.pdf]

Cost-effectiveness of Pharmaceutical-based Pandemic Influenza Mitigation Strategies

Technical Appendix 2

Parameters: base-case and sensitivity range

Parameter	Base-case	Distribution	Distribution parameters	Source
Population:				
General population (age in years)		–	–	*(1)*
0–19	5,513,878			
20–64	12,744,215			
≥65	2,759,129			
Total	21,017,222			
Average life-expectancy (years)		–	–	*(2)*
0–19	72.1			
20–64	41.9			
≥65	13.0			
Percentage of population employed		–	–	*(3)*
15–19	47.3%			
20–64	74.8%			
≥65	9.7%			
Disease:				
R_0	1.7	Triangular	Min: 1.1, Max: 2.3, Mean: 1.7	*(4–6)*
Percentage of those infected who are symptomatic	50%	Beta	α=11, β=11	*(7)*
Latent period	1 day	Gamma	Shape: 25, Scale: 0.04	*(8)*
Infectious period	2 days	Gamma	Shape: 40, Scale: 0.05	*(8)*
Relative infectiousness of asymptomatic individuals	66.7%	Beta	α=6, β=3	*(9, 10)*
Percentage of individuals clinically infected who seek primary care		Beta		*(11)*
Strategy 1/3	50%		α=20, β=20	
Strategy 2/4	80%		α=24, β=6	
Percentage of primary care general practitioner (vs emergency department)	80%	–	–	
Case-hospitalization rate		Beta	α=3, β=117	
0–19	1.875%			

20–64	2.5%			
≥65	5%			
Case-fatality rate		Beta	α=5, β=495	
0–19	0.75%			
20–64	1%			
≥65	2%			
Time to pandemic	5 years	Exponential		
Number of days absent from work for individuals clinically infected	2.6	Gamma	Shape: 8, Scale: 0.325	*(12)*
Vaccine and antiviral drugs				
Efficacy against infection				
Pre-pandemic vaccine				
1 dose	5%	(in proportion to 2nd dose efficacy)		
2 dose	40%	1-Efficacy ~ Lognormal	exp(μ)=0.6, σ=0.1	
Matched vaccine				*(13)*
1 dose	40%	(in proportion to 2nd dose efficacy)		
2 dose	80%	1-Efficacy~ Lognormal	exp(μ)=0.2, σ=0.1	
Booster to prepandemic	80%	1-Efficacy~ Lognormal	exp(μ)=0.2, σ=0.1	
Vaccine efficacy in ≥65 age group	50% of other age-groups	–	–	*See main text*
Antiviral Prophylaxis	-	-	-	*(14)*
Efficacy against infection	70%	1-Efficacy ~Lognormal	exp(μ)=0.3, σ=0.1	
Efficacy against transmission	60%	1-Efficacy ~Lognormal	exp(μ)=0.4, σ=0.1	
Antiviral Treatment				*(15)*
Reduction in hospitalization	59%	1-Efficacy~ Lognormal	exp(μ)=0.41, σ=0.2	
Reduction in mortality	59%	1-Efficacy~ Lognormal	exp(μ)=0.41, σ=0.2	
Percentage receiving antiviral treatment within 48 hours	80%	Beta	α=8, β=2	
Percentage of contacts of clinical cases receiving effective post-exposure antiviral prophylaxis	30%	Beta	α=6, β=14	
Percentage of infections resistant to antiviral drugs	10%	Beta	α=9, β=1	
Shelf life of vaccine	3 years	Gamma	Shape: 3, Scale: 1	
Shelf life of antiviral	5 years	Gamma	Shape: 20, Scale: 0.25	
Size of antiviral stockpile:		–	–	
Strategies 2/4	41% of population			
Strategies 1/3	1% of population			
Vaccine coverage	80%	Beta	α=8, β=2	*(16)*
Matched vaccine 1st dose timing- Days given post pandemic infection in Australia	180 days	Normal	μ=180, σ=30	
Pre-pandemic vaccine 1st dose timing- Days after pandemic infection in Australia	0 days	Normal	μ=0, σ=30	
Costs:				

Vaccine pre-pandemic and matched (per dose)	$12.00	Lognormal	exp(μ)=12, σ=0.08	
Administration (per dose)	$11.60	Lognormal	exp(μ)=11.60, σ=0.3	*(17)*
Antiviral treatment (per course)	$32	Lognormal	exp(μ)=32, σ=0.03	
Administration prophylaxis (per course)	$11.60	Lognormal	exp(μ)=11.60, σ=0.3	
Storage cost (per year)				
Antiviral per course	$0.5	Gamma	Shape: 1.5, Scale: 1/3	
Vaccine per dose	$1	Gamma	Shape: 1.5, Scale: 2/3	
General practitioner visit	$33.32	Lognormal	exp(μ)=33.32, σ=0.09	*(18)*
Emergency department visit	$72.35*	Lognormal	exp(μ)=72.35, σ=0.09	*(19)*
Hospitalization (per day)	$860.85	Lognormal	exp(μ)= 860.85, σ=0.09	*See main text*
Average hospitalization length of stay in days				*(20)*
0–19	3.0	Gamma	Shape: 4, Scale: 0.75	
20–64	5.1	Gamma	Shape: 6.8, Scale: 0.75	
≥65	8.0	Gamma	Shape: 10.67, Scale: 0.75	
Lost work day	$161	Lognormal	exp(μ)=161, σ=0.05	*(21)*
Discounting rate		–	–	*(22)*
Costs	5%			
Effects	5%			

*Standardized to 2005 Australian $ using consumer price indices

References

1. Australian Bureau of Statistics. Australian demographic statistics (ABS catalogue number: 3101.0) Canberra: Australian Bureau of Statistics; 2007.

2. Australian Bureau of Statistics. Life tables, Australia, 2005-2007 (ABS catalogue number: 3302.0.55.001). Canberra: Australian Bureau of Statistics; 2008.

3. Australian Bureau of Statistics. Australian labour market statistics (ABS catalogue number: 6105.0). Canberra: Australian Bureau of Statistics; 2009.

4. Bootsma MC, Ferguson NM. The effect of public health measures on the 1918 influenza pandemic in U.S. cities. Proc Natl Acad Sci U S A. 2007;104:7588–93. PubMed DOI: 10.1073/pnas.0611071104

5. Caley P, Philp DJ, McCracken K. Quantifying social distancing arising from pandemic influenza. J R Soc Interface. 2007 Oct 4.

6. Vynnycky E, Edmunds WJ. Analyses of the 1957 (Asian) influenza pandemic in the United Kingdom and the impact of school closures. Epidemiol Infect. 2008;136:166–79. PubMed

7. Department of Health. Swine flu: UK planning assumptions. London: Department of Health; 2009.

8. Halloran ME, Ferguson NM, Eubank S, Longini IM Jr, Cummings DAT, Lewis B, et al. Modeling targeted layered containment of an influenza pandemic in the United States. Proc Natl Acad Sci U S A. 2008;105:4639–44. PubMed DOI: 10.1073/pnas.0706849105

9. Longini IM Jr, Halloran ME, Nizam A, Yang Y. Containing pandemic influenza with antiviral agents. Am J Epidemiol. 2004;159:623–33. PubMed DOI: 10.1093/aje/kwh092

10. Longini IM Jr, Nizam A, Xu S, Ungchusak K, Hanshaoworakul W, Cummings DA, et al. Containing pandemic influenza at the source. Science. 2005;309:1083–7. PubMed DOI: 10.1126/science.1115717

11. AIHW GP Statistics and Classification Unit. SAND abstract No 27 from the BEACH program: Prevalence and management of influenza 2000.

12. AIHW GP Statistics and Classification Unit. SAND abstract No 9 from the BEACH program: Influenza and absenteeism. 2000.

13. Demicheli V, Di Pietrantonj C, Jefferson TO, Rivetti A, Rivetti D. Vaccines for preventing influenza in healthy adults. Cochrane Database Syst Rev. 2007; (2):CD001269. PubMed

14. Halloran ME, Hayden FG, Yang Y, Longini IM Jr, Monto AS. Antiviral effects on influenza viral transmission and pathogenicity: observations from household-based trials. Am J Epidemiol. 2007;165:212–21. PubMed DOI: 10.1093/aje/kwj362

15. Kaiser L, Wat C, Mills T, Mahoney P, Ward P, Hayden F. Impact of oseltamivir treatment on influenza-related lower respiratory tract complications and hospitalizations. Arch Intern Med. 2003 07/28/;163(14):1667-72.

16. Australian Institute of Health and Welfare. 2004 Adult vaccination survey: summary results (AIHW catalogue number: PHE 56). Canberra: Australian Institute of Health and Welfare; 2005.

17. Municipal association of Victoria. Cost of Victorian local government immunisation services. Melbourne: Municipal association of Victoria; 2004.

18. Newall AT, Scuffham PA. Influenza-related disease: The cost to the Australian healthcare system. Vaccine. 2008;26:6818–23. PubMed DOI: 10.1016/j.vaccine.2008.09.086

19. Commonwealth Department of Health and Ageing. Manual of resource items and their associated costs Canberra: Commonwealth Department of Health and Ageing; 2002.

20. Australian Institute of Health and Welfare. AIHW National Hospital Morbidity database (http://www.aihw.gov.au/hospitals/datacubes/datacube_pdx.cfm): Influenza and pneumonia 2007-08.

21. Australian Bureau of Statistics. Average Weekly Earnings, Australia (ABS catalogue number: 6302.0). Canberra: Australian Bureau of Statistics; 2005.

22. Commonwealth Department of Health and Ageing. 2006 Guidelines for the Pharmaceutical Industry on Preparation of Submissions to the Pharmaceutical Benefits Advisory Committee (PBAC) Version 4.1. Canberra: Commonwealth Department of Health and Ageing; 2006.
